# The *DPYSL2* gene connects mTOR and schizophrenia

**DOI:** 10.1038/tp.2016.204

**Published:** 2016-11-01

**Authors:** X Pham, G Song, S Lao, L Goff, H Zhu, D Valle, D Avramopoulos

**Affiliations:** 1 McKusick-Nathans Institute of Genetic Medicine, Johns Hopkins University School of Medicine, Baltimore, MD, USA; 2 Predoctoral Training Program in Human Genetics, McKusick-Nathans Institute of Genetic Medicine, Johns Hopkins University School of Medicine, Baltimore, MD, USA; 3 Department of Pharmacology and Molecular Sciences, Baltimore, MD, USA; 4 University of Maryland, College Park, MD, USA

## Abstract

We previously reported a schizophrenia-associated polymorphic CT di-nucleotide repeat (DNR) at the 5′-untranslated repeat (UTR) of *DPYSL2*, which responds to mammalian target of Rapamycin (mTOR) signaling with allelic differences in reporter assays. Now using microarray analysis, we show that the DNR alleles interact differentially with specific proteins, including the mTOR-related protein HuD/ELAVL4. We confirm the differential binding to HuD and other known mTOR effectors by electrophoretic mobility shift assays. We edit HEK293 cells by CRISPR/Cas9 to carry the schizophrenia risk variant (13DNR) and observe a significant reduction of the corresponding CRMP2 isoform. These edited cells confirm the response to mTOR inhibitors and show a twofold shortening of the cellular projections. Transcriptome analysis of these modified cells by RNA-seq shows changes in 12.7% of expressed transcripts at a false discovery rate of 0.05. These transcripts are enriched in immunity-related genes, overlap significantly with those modified by the schizophrenia-associated gene, *ZNF804A,* and have a reverse expression signature from that seen with antipsychotic drugs. Our results support the functional importance of the *DPYSL2* DNR and a role for mTOR signaling in schizophrenia.

## Introduction

*DPYSL2* encodes a cytosolic protein best known as collapsin response mediator protein 2 (CRMP2), a member of a five-gene family named for their involvement in axonal growth cone collapse. Mammals express three *DPYSL2* transcripts, A, B and C, each differing in an alternative first exon. Nearly all published literature, including this report, has focused on the B transcript, which we henceforth refer to as *DPYSL2B* with the corresponding protein isoform CRMP2B. The 5′-untranslated repeat (UTR) of *DPYSL2B* contains a 5'-terminal oligopyrimidine (5'-TOP) tract, a consensus sequence found in genes regulated translationally by the mammalian target of Rapamycin (mTOR) pathway.^1, 2^ The 5'-TOP sequence is characterized by a cytosine preceding a tract of pyrimidines.^3, 4^ *DPYSL2B* is primarily expressed in the central nervous system and has vital functions in neuronal development.^5, 6, 7, 8^ The CRMP2 protein interacts with other proteins to stabilize microtubules, promote neurite outgrowth and modulate signaling processes in the central nervous system.^9, 10, 11, 12^ The CRMP2 interactome has been previously explored,^13^ and according to Panther Bioinformatics^14^ it is strongly enriched in microtubular proteins (sevenfold, corrected *P*=2 × 10^−3^) and proteins involved in microtubular processes (sixfold, corrected *P*=0.02). Altering the expression of Crmp2 in cultured rat hippocampal neurons significantly affects axonal growth.^15, 16^ Further, multiple proteomic studies report aberrant expression of CRMP2 in the brains of schizophrenics.^17, 18, 19, 20^ Most recently, a study showed downregulation of the Crmp2 protein in the prefrontal cortex and hippocampus of prenatally stressed rats.^21^ The study also reported two single-nucleotide polymorphisms associated with schizophrenia (SZ) susceptibility, further suggesting a role for *DPYSL2* in this disease.^21^

The mTOR complex 1 (mTORC1) is a serine/threonine kinase complex that controls protein synthesis by phosphorylating downstream target effectors. The complex owes its name to the immunosuppressant drug Rapamycin, which strongly inhibits mTORC1 kinase activity. Canonical activation of mTORC1 results from an activation cascade of upstream proteins, including receptor tyrosine kinases, PI3K and AKT. Once active, mTORC1 stimulates its downstream effector proteins, S6 and 4E-BP, which, respectively, increase ribosome biogenesis and mRNA translation.^22^ Activation of mTOR boosts overall cell growth and metabolism, making it a well-known target for cancer therapy. Increasingly however, mTOR has been linked to the development of the central nervous system, neuronal growth, maintenance and proliferation.^23, 24^ Perturbation of mTOR signaling affects neurotransmitters involved in SZ, such as serotonin, glutamate and their receptors, which has led to the hypothesis that this pathway could have a significant role in SZ.^24^

Family, twin and adoption studies show strong genetic contribution to risk for SZ,^25, 26^ and in a linkage analysis covering ~70% of the genome in 57 multiplex pedigrees we previously identified region 8p21–22 as a candidate SZ susceptibility locus (*P*=0.0001).^27^ This result was replicated by many others in different populations,^28, 29^ and we followed it up with a targeted association study in an Ashkenazi Jewish population.^30^ We found that the most significant single-nucleotide polymorphism (rs12155555) was located ~5 kb upstream of *DPYSL2B,* and the strongest association signals from the fine mapping were located within or flanking the *DPYSL2B* gene.^30^ Subsequently, we identified multiple functional sequence variants in *DPYSL2B,* including a polymorphic CT di-nucleotide repeat (DNR) variant located within the gene's 5'-TOP tract.^31^ In Caucasians, this DNR most often comprises 11 CT repeats (11DNR). The next most common allele caries 13 CT repeats (13DNR) and previously, in a small sequencing study, we found it at a frequency of ~14.5% in 93 healthy Caucasians and 29% in 46 Caucasians with SZ.^32^ Because of the nature of this polymorphism, it is not typed or captured well through linkage disequilibrium in genome-wide studies. Using dual luciferase and polysome-profiling assays we showed that the 13DNR risk allele significantly reduced expression at the translation level. Further, we reported a dose-dependent response of both DNR alleles to Rapamycin, an allosteric inhibitor mTOR. The results of that work suggested that the *DPYSL2B* 5'-TOP sequence responds to mTOR signaling, and this response is altered by the 13DNR allele.^31^

Here we report on experiments that extend and strengthen these results. First, we characterize the 5′-UTR of *DPYSL2B* for interaction with specific proteins, including mTOR effectors. Using CRISPR/Cas9-modified human embryonic kidney-derived cells (HEK293), we show that this naturally occurring DNR variant has strong effects on *DPYSL2B* regulation in its native context in the genome, the longer repeat leading to significant reduction of the corresponding CRMP2 isoform. This change is accompanied by striking shortening of the natural HEK293 projections. Finally, we observe that significant changes in the transcriptome point to pathways implicated in SZ overlap with changes attributed to other SZ susceptibility genes, and oppose those observed by exposure to antipsychotics.

## Materials and methods

### Protein interaction analyses

To study the differential binding of proteins to the DNR alleles, we performed protein microarray analysis as previously described^33^ (Supplementary Materials). Briefly fluorescently labeled RNA probes were synthesized for the 11 and 13 DNRs and hybridized in duplicates to protein microarrays of >4,000 proteins each printed in duplicate. After hybridization, the protein chips were scanned and measurements were background corrected and Z-normalized. Positive hits were defined as proteins with Z-score > 9 and if the difference between the two alleles was less than two standard deviations they were considered to bind both alleles, otherwise to bind preferentially to either the 11 or 13 DNR allele. Confirmation experiments were performed using electrophoretic mobility shift assays under standard protocols^34^ (Supplementary Materials). Briefly the same 11 and 13DNR RNA probes as above were 5' end-labeled with radioactive γ–ATP-32 and radioactivity was quantified in a scintillation counter and normalized to 4,000 counts per minute. They were then incubated with commercially purified candidate binding proteins on ice for 20 minutes, electrophoresed in TBE through a 5% polyacrylamide gel and autoradiographed

### CRISPR/Cas9 targeting

To study the effect of the DNR in its genomic context, we used the CRISPR/Cas9 system to create two isogenic cell lines with the 11 and 13DNR in HEK293 cells, which normally carry the 11 repeat DNR. HEK293s are human cells widely used in neuroscience with many technical advantages including relatively fast growth and high transfection efficiency. Reports suggest that they may be of neuronal origin,^35^ despite been derived from kidney, and they have natural projections, which allow us to test effects on the cytoskeleton as we expect for this microtubular gene. Using the online tool provided by the Zhang Lab at MIT (crispr.mit.edu), we designed three guide RNAs and a repair template to insert two CT repeats at the DNR region (Supplementary Materials). We used the IDT gBlock system to synthesize guide RNA constructs.

We used lipofectamine (ThermoFisher, Waltham, MA, USA, cat #11668027) to co-transfect the guide RNA, the repair template and a GFP-tagged Cas9 nuclease (Addgene, Cambridge, MA, USA, cat #44719) to HEK293 cells. Post transfection, the cells underwent FAC sorting into 96-well plates (one cell per well). The single cells were then expanded into clonal colonies, the DNA of which was used for sequencing to identify homozygotes for each allele.

We then selected 17 gene loci as candidates for off-target effects predicted by the online tool developed by the Zhang Lab at MIT to check for off-target effects. Using primers (Supplementary Materials) designed to capture and Sanger sequence 100–200 bp of these candidate loci, we identified no known off-target effects of the gene-editing scheme.

### Western blot

Cell lysates were obtained from the four 13DNR homozygote clones and four targeted 11DNR control clones, assayed with a colorimetric assay and normalized to 20ug total protein. After heating to 95C for 5 minutes they were electrophoresed in 15% polyacrylamide and transferred to a Polyvinylidene fluoride membrane. The membrane was blocked in 5% powder milk and probed with rabbit anti-CRMP2 antibody at 1:5000 (Sigma, St. Louis, MO, USA, cat. # C2993) and mouse anti-GAPDH at 1:4,000 (AbCam, Cambridge, UK cat. # ab9485). Following washes they were probed with mouse (AbCam cat. # ab6728) and rabbit (ThermoFisher, cat. # 656120) HRP-conjugated secondary antibodies at 1:10,000 in 1% milk washed, and visualized by chemiluminescence and autoradiography. For Rapamycin exposures, we grew cells in standard media containing 30nM Rapamycin (SelleckChem, Houston, TX, USA, cat. # S1039) for 24 hours. After drug exposure, we obtained the cell lysates as described above. For more information see Supplementary Materials.

### Real-time qPCR

RNA was obtained from the selected CRISPR-edited clones using an RNeasy Mini Kit (Qiagen, Valencia, CA, USA, cat. # 74104) and reverse-transcribed using qScript cDNA Supermix (Quanta Biosciences, Beverly, MA, USA, cat. # 84033). We performed qPCR using PerfeCTa SYBR Green FastMix (Quanta Biosciences #95072) and primers for B-actin and DPYSL2. Samples were run on the 7900HT Sequence Detection System by Applied Biosystems. At the end of the run, a melting curve analysis confirmed that only a single product was amplified.

### Immunofluorescence and imaging

Targeted HEK293 cells carrying the homozygous forms of the 11 and 13 DNR alleles were grown on Poly-D Lysine (Sigma, cat. #P6407) coated coverslips in 12-well plates in standard media until 75-85% confluent. They were then washed twice with 1x passive buffered saline, fixed in paraformaldehyde, washed again and incubated with 1mg/ml wheat germ agglutinin conjugated with Alexa Fluor 488 (ThermoFisher, cat. # W11261). The coverslips were mounted on slides with media containing DAPI stain and confocal microscope images were acquired. For more culture and labeling details see Supplementary Materials. Eighteen slides (nine from each cell group) were randomized and blinded before imaging. We had four clones per genotype and three slides per clone. We collected eight images from each slide. The images were analyzed for cellular projection length differences using ImageJ (National Institutes of Health, Bethesda, MD, USA, https://imagej.nih.gov/ij/).

### RNA sequencing

We obtained cell pellets for RNA-seq from the four 13DNR homozygous edited clones, and eight targeted 11DNR control clones. RNA was extracted from the cell pellets, quantified and normalized to 500ng per sample and the samples were then sent to the Hopkins Core Facility for sequencing where an Agilent BioAnalyzer was used to ensure RNA quality (RIN >8.5) and Illumina's TruSeq RNA v2 protocol was used to generate libraries after RiboMinus (ThermoFisher) ribosomal RNA depletion. The resulting library insert size was 120-200bp with a median size of 150bp. DNA sequencing was performed on an Illumina HiSeq 2500 instrument using standard protocols for paired-end 100bp sequencing. For more details on RNA exraction, library generation and sequencing see Supplementary Materials.

### RNA-seq data analysis

Forward and reverse raw reads separated by 12 barcodes were received from our core facility showing no problems in data quality according to the accompanying FastQC reports (http://www.bioinformatics.babraham.ac.uk/ projects/fastqc). Analysis was performed using the Tuxedo suite of programs including tophat for read mapping, cufflinks for transcript assembly and cuffdiff for transcript abundance comparisons as described.^36^ Following the Tuxedo pipeline, the output was further processed using the R Bioconductor package CummeRbund (http://bioconductor.org) for analysis and graphical visualization.

Our raw and processed expression data have been deposited to GEO under accession number GSE82237.

## Results

### The *DPYSL2B* 5′-UTR DNR alleles differentially bind TORC1 effectors and HuD

To identify proteins binding to the 5'-TOP sequence of *DPYSL2B*, we used protein microarrays we have previously developed.^33^ We found exclusive binding of the 11DNR to the following five proteins: CHAMP1, a chromosome alignment phosphoprotein necessary for mitotic division; CAST, a calpain inhibitor involved in proteolysis; LMOD3, a leiomodin family member involved in skeletal muscle organization; TCEAL5, an X-linked transcription elongation factor; and ELAVL4 a ribosomal binding protein with exclusive neuronal expression.^37^ *ELAVL4*, also known as *Human Antigen D (HuD)*, is a member of a four-gene ribosomal binding protein family. Its known functions include mRNA transport from the nucleus to the cytoplasm and the triggering of neuronal differentiation.^38^ Like *DPYSL2B*, *ELAVL4* is regulated by the mTOR pathway and promotes dendritic branching.^39^

**Figure 1:**
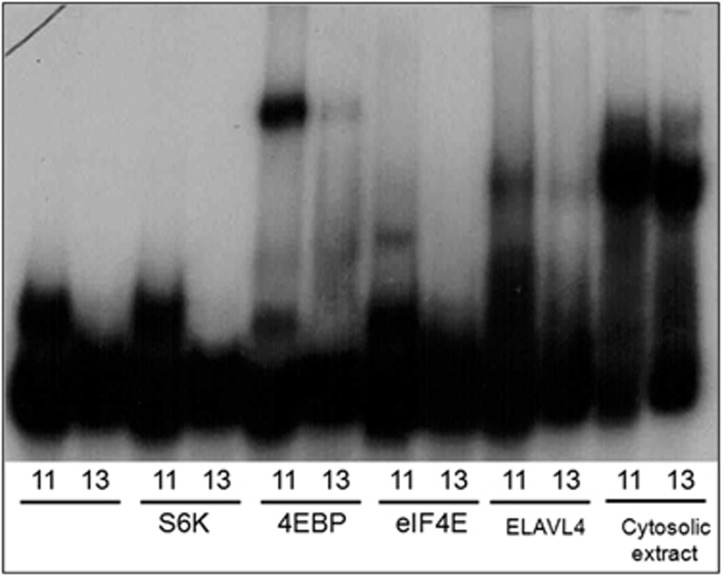
Audiograph of electrophoretic mobility shift assay using labeled RNA probes for the 11 and 13DNR DNR alleles, and purified candidate-binding proteins. Results confirm binding for 4E-BP and elF4E and ELAVL4, seen as a shift up from the unlabeled oligos. In all cases, the binding was weaker, or eliminated for the 13 repeat risk allele. (The small upward shift of the 11DNR-unbound oligo band is likely due to a secondary structure, as it can be reduced or eliminated with heat treatment. See Supplementary Materials.) DNR, di-nucleotide repeat.

Electrophoretic mobility shift assays confirmed these results (Figure 1) and showed differential binding of the DNR alleles to two additional mTOR pathway proteins 4E-BP and eIF4E (Figure 1 and Supplementary Figure 1).

### The 13DNR allele expresses lower levels of the corresponding protein isoform in HEK293 cells, further reduced by blocking mTORC1

To test whether the differences we observed between the DNR alleles in reporter assays also apply to the *DPYSL2B*B transcript, we introduced the 13DNR allele into HEK293 cells using CRISPR/Cas9 genome-editing technology.^40^ After targeting, we obtained 112 successfully transfected HEK293 cell clones, flow-sorted for the presence of the co-transfected GFP tag. Of these, four (3.6%) were 13DNR homozygotes and three (2.7%) were heterozygotes (Supplementary Figure 2). The remaining clones were not modified at the target site, and were used as 11DNR controls. We randomly selected eight of these controls and the four homozygous 13DNR clones for further study. Sequencing at the 17 significant candidate sites identified no off-target mutations.

**Figure 2:**
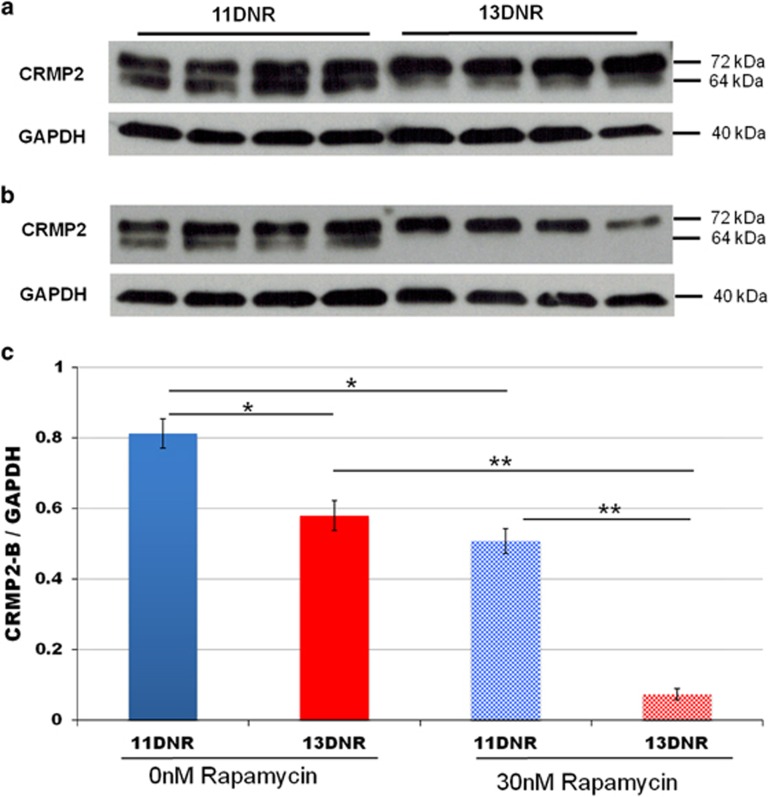
(**a**) Western blots of targeted clones show significant differences in collapsin response mediator protein B2 (CRMP2B) levels (64 kDa) between the two isogenic cell groups. (**b**) In the presence of 30 nM Rapamycin, CRMPZB is reduced for both cell groups, but most dramatically in the 13PNR cells. (**c**) Quantification of western blots using the ImageJ software. *P<0.001; **P<2 × I0^−5^. DNR, di-nucleotide repeat.

Western blot on four DNR11 and four DNR13 clones using a CRMP2 antibody recognizing all transcripts showed significant differences between the isogenic cell groups (Figure 2a and c). CRMP2B, migrating at 64 kDa, was significantly reduced in 13DNR cells as compared with 11DNR cells, both relative to CRMP2A migrating at 72 kDa (*P*=6.5 × 10^−6^) and in absolute terms after normalization to GAPDH (*P*=0.0077; Figure 2a and c). The absolute levels of CRMP2A were not affected (*P*=0.826).

After exposure to 30 nM Rapamycin, levels of CRMP2B protein in the 13DNR clones were reduced by nearly eightfold (*P*=2.8 × 10^−5^) compared with a 1.6-fold change for the 11DNR clones (*P*=0.0014; Figure 2b, c). The reduction recapitulates our previous observations in *DPYSL2B* promoter constructs driving luciferase.^31^ Overall, the results suggest that the 13DNR allele has a weaker response to mTOR signaling, and is more sensitive to mTOR inhibition. In contrast to the protein results, the transcript profiles for *DPYSL2B* were not significantly different when measured by *DPYSL2B*-specific RT-PCR between the 11 and 13DNR cells, supporting that the DNR primarily affects translation as expected for mTOR signaling.

### HEK293 cells homozygous for the 13DNR alleles develop fewer and shorter projections than their 11DNR counterparts

**Figure 3:**
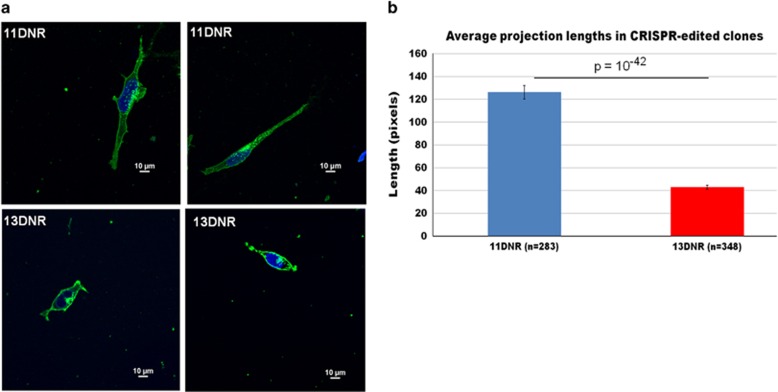
(**a**) Representative images of CRISPR-edited cells show morphological differences between the 11 and 13DNR cell groups. Cells stained tor wheat germ agglutinin (green) and nuclei (blue). (**b**) Quantification of projection lengths between the two isogenic cell groups confirms a significant difference, with the 13DNR risk allele showing shorter projections. DNR, di-nucleotide repeat.

HEK cells naturally show neurite-like projections and have been suggested to have a neuronal lineage because of the original transformation methodology.^35^ They have also been found to express multiple neuron-specific markers.^35^ Following the introduction of the 13DNR, we observed that the HEK293 projections appeared shorter in the homozygous 13DNR cells (Figure 3a). This observation was confirmed by blinded projection length measurements and comparisons between homozygous 11DNR cells (*n*=283) and homozygous 13DNR cells (*n*=348, *P*=10^−42^) using imageJ.^41^ The estimated length difference was approximately twofold (Figure 3b).

### The 13DNR homozygote HEK293 cells show striking transcriptome-level differences particularly for immune system genes

**Figure 4:**
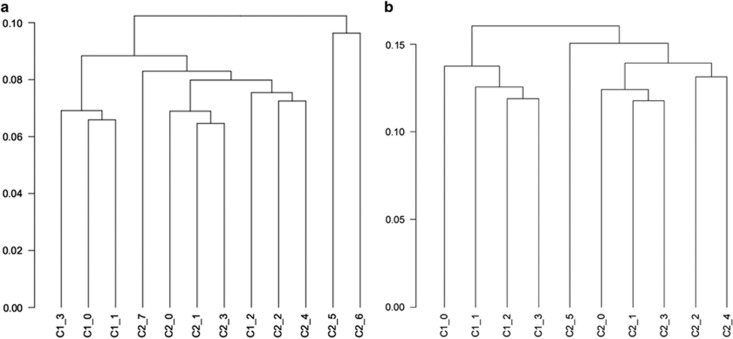
Dendrograms based on all genes. C1 is 13DNR and C2 is 11DNR. (**a**) Two 11DNR samples (C2_5 and C2_6) cluster together and separate early from all other samples, which together with the increased variance in the group, suggest that they are outliers. (**b**) After removing the outliers, the variance was restored; the first branching of the dendogram now separates the four 13DNR (C1) in one group and six 11DNR (C2) in the the other. Note that the numbering of the cell lines has now changed—C2_5 corresponds to previously C2_7. DNR, di-nucleotide repeat.

The cell's transcriptional output is the first level of response to change, be it genetic or environmental. Having shown that the DNR variant has a strong effect on isoform abundance, we then asked whether this change is important for the cell's homeostasis as reflected in its transcriptome, and whether the transcriptome changes support a role in disease. We performed RNA sequencing (RNA-seq) of the two isogenic cell groups acquiring ~20 million paired reads for each of the 12 individually targeted clones (4 × 13DNR and 8 × 11DNR). The reads showed 85–90% unique alignment to the human genome. Initial quality-control showed that, despite all samples having similar fragments per kilobase per million (FPKM) distributions, the 11DNR samples showed significantly higher variance across FPKM levels (Supplementary Figure 3A). A dendrogram based on all genes identified two 11DNR samples (C2_5, C2_6) that separated early from all other samples and clustered together, suggesting they are outliers (Supplementary Fig. 3B). We considered that this may be either due to undetected off-target editing or to the chromosomal instability of this neoplastic cell line. Removing them restored the variance of the remaining six 11DNR samples to levels similar with the 13DNR. A new dendrogram using all genes on the new set of samples clustered the six 11DNR and four 13DNR samples separately, showing that the genome-wide expression profile differences were driven mostly by the *DPYSL2B* DNR genotype (Figures 4a and b).

From the 13 548 genes that were expressed in these cells, 1730 (12.7%) showed expression differences (false discovery rate<0.05) between the 11DNR and 13DNR groups. Of those, 802 were higher in 13DNR and 928 in 11DNR. We used PANTHER bioinformatics to test whether the up- and downregulated genes showed enrichment for membership to different functional categories compared with the set of expressed genes. The most significant enrichment in biological processes was for genes expressed lower in the 13DNR that were often immune system process genes (2.04-fold, *P*=2.6 × 10^−7^). This mirrors enrichments seen in SZ genes by both genome-wide association study (GWAS)^42^ and proteomics analyses.^43, 44, 45^ Genes expressing higher in 13DNR were strongly enriched for those encoding RNA-binding proteins (2.6-fold, *P*=5.25 × 10^−10^). The complete results at *P*<0.01 are shown in Table 1.

### The genes changing as a result of the 13DNR overlap significantly with genes changing by perturbing another SZ gene, *ZNF804A*

A previous study by Chen *et al.*^46^ used an approach similar to ours on another SZ-associated gene, *ZNF804A.* That study performed RNA-seq analysis after a knockdown approach to reduce the expression of *ZNF804A* in neural progenitor cells derived from induced pluripotent stem cells. Reduced expression of *ZNF804A* has been reported to be the consequence of the SZ-associated allele of single-nucleotide polymorphism rs1344706 in this gene.^47^ Despite significant differences between the studies, when we compared the reported up- or downregulated genes with ours, we found highly significant concordance and overlap. Specifically, of 57 genes whose expression was significantly altered, 45 were in the same direction for both studies (binomial test *P*=6.5 × 10^−6^). The overlap was significantly more than expected by chance for genes that were expressed higher in the SZ risk condition, with 37 genes in common, more than twice the expected 16 (Fisher's exact test *P*=1.6 × 10^−6^), but not in those expressed lower.

### The transcriptome signature of the 13DNR homozygote HEK293 cells is opposite to antipsychotics

The Connectivity Map (cmap) is a collection of genome-wide expression data from human cell lines treated at varying concentrations in culture with 1309 different bioactive small molecules, 18 of which are antipsychotics. Pattern-matching computational tools integrated in the cmap website (www.broadinstitute.org/cmap) allow the user to upload any gene signature (a set of up- and downregulated genes) and compare it with the signatures of these small molecules for positive or negative correlations. We did this analysis for the transcriptional signature of the 11DNR/13DNR variant and found that 3 of the top 10 cmap signatures most significantly matching the 13DNR group (all *P*<2 × 10^−5^) were the antipsychotics Thioridazine, Trifluoperazine and Prochlorperazine. All three had complimentary (that is, opposite) signatures to the 13DNR allele. Interestingly, Rapamycin, the mTOR inhibitor, was also in the top 10 signatures with complimentary signature to the 13DNR risk allele.

## Discussion

We have demonstrated that the DNR variant in the 5′-UTR of the *DPYSL2B* transcript has strong effects on the expression of the corresponding protein isoform, and provided additional evidence that this is likely due to changes in its response to mTOR signaling. We found reduced binding to the 13DNR risk allele of two downstream mTOR effectors, 4E-BP and eIF4E, which may mediate these changes. In eukaryotes, phosphorylated 4E-BP releases eIF4E, allowing the de-repressed factor to initiate cap-dependent translation.^48, 49^ We also found that the binding of ELAVL4 to the 11DNR and 13DNR alleles in the 5'-TOP sequence of *DPYSL2B* is markedly different. The striking similarities in CRMP2 and ELAVL4 functions in neurons and their common regulation by mTOR make this an interesting lead for further research.

The mTOR pathway promotes cell growth and division, and is widely known for its involvement in cancer.^50, 51^ Mounting evidence, however, also implicates mTOR in brain development and plasticity, with aberrant signaling contributing to neurodevelopmental disorders including SZ.^24, 52, 53^ Our results support this hypothesis. We show that the translation of *DPYSL2B*, a candidate SZ susceptibility gene involved in axonal growth and neuronal development, is regulated by mTOR. The response to Rapamycin, the binding of mTOR effectors and the transcriptome changes in the presence of the weaker binding allele not only demonstrate this but also provide candidate mechanisms for the connection to disease.

The link between mTOR and SZ risk is particularly interesting because it provides a potential explanation for environmental disease risk factors. The importance of starvation in SZ risk has been strongly established by studies in the Dutch and Chinese populations that experienced severe famines in the early to mid Twentieth century.^54, 55^ In these populations, prenatal famines led to a twofold increased risk for SZ. The mTOR pathway is highly sensitive to external environmental factors and, in particular, the availability of nutrients.^56^ Starvation or otherwise malnutrition negatively affects the activity of this pathway, and could conceivably increase SZ risk through changes in the regulation of genes involved in nervous system development such as *DPYSL2B*.

We used genome editing by CRISPR/Cas9, and created two isogenic cell lines differing genetically only in the DNR (CT) repeat length at the 5′-UTR of *DPYSL2B*. We observed that 13DNR homozygotes produced significantly less CRMP2B protein. Consistent with the known functions of CRMP2B, they also had significantly shorter cellular projections. The transcriptome differences between 11 and 13DNR cells showed that many genes are affected by this genetic change, presumably as a result of the change in CRMP2B production. These genes (Supplementary Table 1) provide important clues for unraveling the pathways involved in the function of CRMP2 and its possible connections to disease.

In previous work,^32^ we found in a small sample that the 13DNR allele frequency was twice as high in SZ patients as in controls. To get a better estimate of the effect in a large sample, we have identified two single-nucleotide polymorphisms (rs367948 and rs445678) that are in imperfect linkage disequalibrium with this repeat, predicting the repeat genotypes in 41 of 42 individuals (SZ patients) with available data (data not shown). In the results of the PGC2 GWAS,^42^ these show a small increase in the risk of 1.1-fold (*P*=0.04–*P*=0.009, respectively, in the expected direction). It is remarkable that a variant with such profound effects on the transcriptome and on cell morphology in cultured cells has such a small effect on the phenotype of the organism. This suggests that strong buffering mechanisms minimize the consequences of the DNR variant in the intact organism. This is not only good news for the carriers of such risk variants, but also for researchers exploring them, as it suggests that despite the small effect on risk typical of most GWAS-identified variants, their effects on isolated cellular models may be strong enough to measure and interpret.

In our transcriptome analyses we found significant changes in 12.7% of transcripts resulting from the 13DNR. The specific genes affected by this variant provided three new lines of support for a connection between the gene and SZ. First, among downregulated genes, the strongest enrichment was for genes involved in immunity. Although not directly intuitive, the immune system has been repeatedly implicated in SZ including the largest GWAS to date,^42^ proteomic studies,^57, 58^ gene expression studies^59^ and reports for a role of infection/inflammation.^60, 61^ Second, we saw a strong overlap and directional consistency between the genes affected by the SZ risk allele in our study and those affected by a similar perturbation in a previously published study of another SZ gene, *ZNF804A*. As more genes are studied, such overlaps should highlight the most important connections between risk variants and the corresponding pathways.

Finally, in a survey of the 1309 pharmaceutical compound-induced expression change signatures in the connectivity map database, three antipsychotics raised to the top significance, all with signatures opposite to that of the 13DNR risk allele. This not only supports our hypothesis connecting the DNR to SZ but, most importantly, it indicates how cellular models might be useful in drug discovery. Other compounds on the top of the list would be interesting for further exploration of potential benefits against psychosis.

More recent findings have provided additional support to the connection between CRMP2, neuronal maintenance and development, and SZ. CRMP2 was recently shown to be involved in autophagy, a cellular recycling process that can be compromised in neurodegeneration. It was shown that the Ester of lanthionine ketimine reduces the suppression mTORC1-mediated autophagy by specifically decreasing mTOR colocalization with LAMP2(+) lysosomes in RG2 cells through a mechanism that involves CRMP2-mediated intracellular trafficking.^62^ In a study of the effects of Docosahexaenoic acid, the most abundant n-3 polyunsaturated fatty acid essential for neuronal development and brain function, it was recently shown that it leads to rapamycin-reversible upregulation of Tau and CRMP2 and increased axon elongation by enhancing their 5'-TOP-dependent translation.^63^ This is in direct agreement with our results on the role and regulation of CRMP2. Finally, Zhang *et al.,*^64^ characterizing a Dpysl2 knockout mouse, found multiple molecular, cellular, structural and behavioral deficits, reminiscent of SZ: reduced long-term potentiation, abnormal *N*-methyl-D-aspartate receptor composition, aberrant dendrite development and defective synapse formation in CA1 neurons. In addition, knockdown of Dpysl2 specifically in newborn neurons led to defects in their development during adult hippocampal neurogenesis. Together with our results and the previous literature, this strengthens the link between SZ and *DPYSL2*, despite the absence of a common variant with significant effect on the risk.

In conclusion, our experiments show that the SZ candidate gene transcript *DPYSL2B* is regulated by the mTOR pathway and this regulation is perturbed by a 5′-UTR SZ-associated DNR, adding support for mTOR's role in psychiatric disease. Further, our study of the transcriptome of genome-edited cells not only confirms the biological significance of the DNR, but it also provides additional links between *DPYSL2B* and SZ genes, pathways and treatments.

## Figures and Tables

**Table 1:** Gene ontology analysis using PANTHER bioinformatics

	*Downrequlated*		*Uprequlated*	
	*Fold enrichment*	P*-value*	*Fold enrichment*	P*-value*
*Biological processes*				
Macrophage activation	3.6	6.43 × 10^−3^	—	—
Immune system process	2.04	2.58 × 10^−7^	—	—
Response to stimulus	1.54	1.47 × 10^−3^	—	—
mRNA splicing via splicesosome	—	—	2.71	3.39 × 10^−4^
mRNA processing	—	—	2.31	8.80 × 10^−^^4^
Nucleobase-containing cmpd metabolic process	—	—	1.28	6.50 × 10^−3^
Metabol ic process	—	—	1.15	6.49 × 10^−3^

*Molecular function*				
Extracellular matrix structural constituent	4.24	6.55 X10^−3^	—	—
Structural molecule activity	1.74	2.22 × 10^−4^	—	—
Receptor activity	1.65	7.91 × 10^−3^	—	—
RNA helicase activity	—	—	4.12	1.28 × 10^−4^
Translation initiation factor activity	—	—	3.15	4.66 × 10^−3^
mRNA binding	—	—	2.88	1.04 × 10^−3^
Helicase activity	—	—	2.78	9.58 × 10^−3^
Translation regulator activity	—	—	2.78	9.58 × 10^−3^
Nucleic acid binding	—	—	2.72	8.76 × 10^−3^
RNA binding	—	—	2.58	5.25 × 10^−10^

*Cellular component*				
Extracellular matrix	—	—	2.56	5.25 × 10^−3^
Extracellular region			2.43	1.56 × 10^−5^
