# Supplementary material for: The DPYSL2 gene connects mTOR and schizophrenia
Source: Transl Psychiatry. 2016 Nov 1;6(11):e933–. doi: 10.1038/tp.2016.204 (PMC5314117; doi:10.1038/tp.2016.204)
Supplement: Supplementary Figures [file tp2016204x1.docx]

**Comment on figure 2 of the main text.**

When we performed EMSAs as shown in figure 2 of the main text we noticed that the 11DNR oligonucleotide was showing an additional band migrating slightly higher than the main band. We performed additional experiments and found that this band could be significantly diminished with heat treatment immediately before loading (unfortunately this step cannot be incorporated in the EMSA experiment), suggesting potential dimers or folding (SFig. 1A). In later experiments and after ordering a new batch of both RNA oligonucleotides we saw the same bands for both 11DNR and 13DNR, and both could also be resolved with heat (SFig. 1B). We used these new oligos in additional EMSA experiments, repeating the initial and adding protein combinations (SFig. 1C). In those experiments the additional bands were present for both alleles, yet there was no difference from our original results. Given these experiments we have excluded that this likely dimer formation or folding does not produce artifacts that could be responsible for the differences we see in binding.


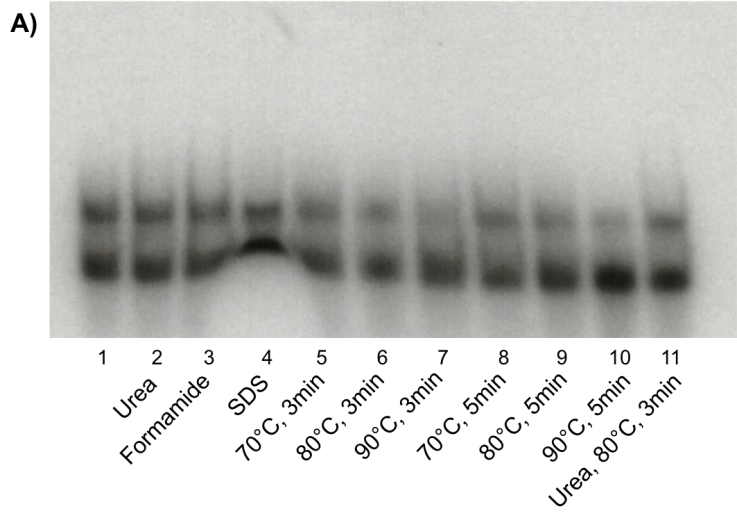

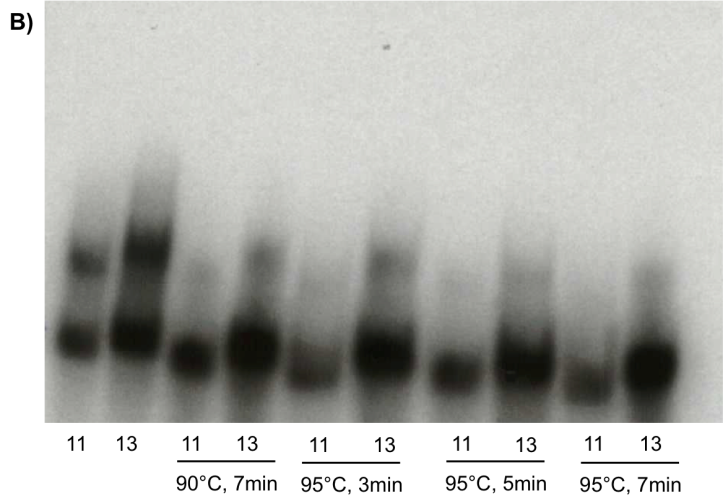


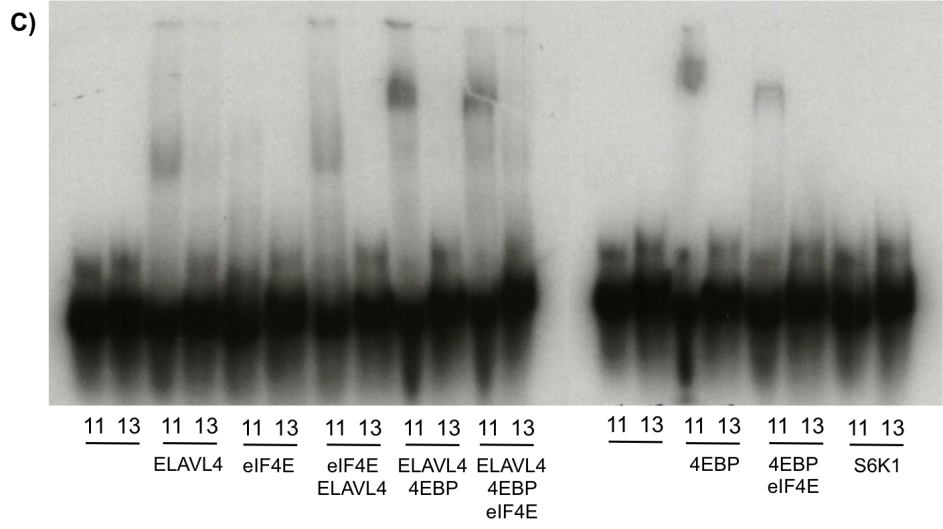


SFigure 1. **(A)** Denaturing conditions at high temperatures for the 11DNR probe shows significant reduction of the secondary band. **(B)** New batch of DNR probes show secondary band for both alleles, which could also be diminished by heat treatment. **(C)** Validation of initial EMSA experiment and protein combinations with new DNR probes. No difference observed from our original results, despite additional band being present for both alleles.


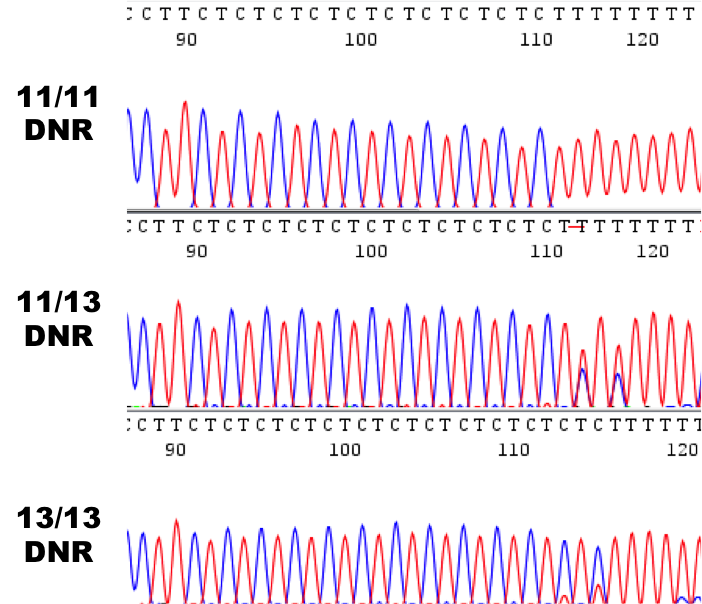


**SFigure 2.** Chromatograph from Sanger sequencing confirming the genotypes of individual targeted clones at the DNR locus. Our targeting scheme yielded 4 clones that were 13DNR homozygous and 3 heterozygotes while the rest were not modified at the target site despite successful transfection, and therefore were appropriate controls.


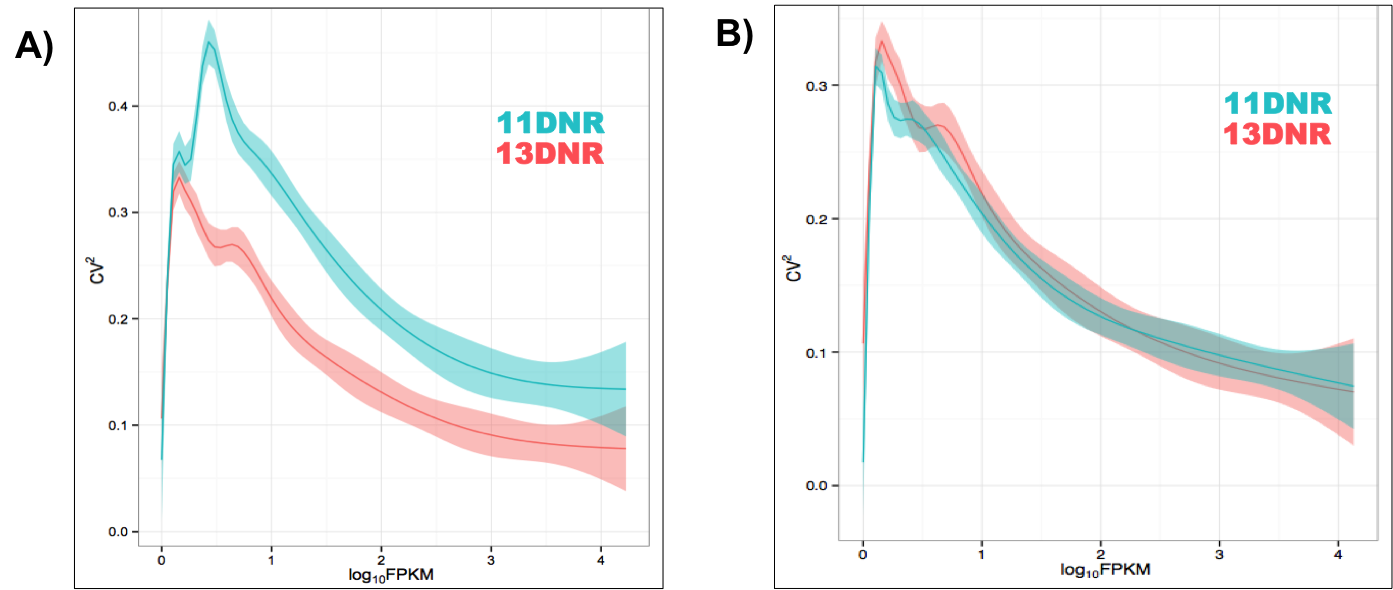


**SFigure 3. (A)** Variance profiles show significantly higher variance across all FPKM levels for the 11DNR. **(B)** Variance profiles for the two groups become similar after removal of the two outliers.
