# Supplementary material for: The DPYSL2 gene connects mTOR and schizophrenia
Source: Transl Psychiatry. 2016 Nov 1;6(11):e933–. doi: 10.1038/tp.2016.204 (PMC5314117; doi:10.1038/tp.2016.204)
Supplement: Supplementary Materials [file tp2016204x2.docx]

**Supplementary materials**

**Supplementary methods**

**Protein microarray analysis.** Fluorescently labeled RNA probes for the 11 and 13 DNRs were synthesized by IDT (See other supplementary information). The probes were hybridized to the protein microarrays in duplicates and as described by Zhu et al. (2007). The microarray chips contained >4,000 proteins, each of which is printed in duplicates, and with multiple sets of positive control proteins. After hybridization, the protein chips were scanned with a GenePix 4000B scanner with 5-µm resolution detection at 635 nm. Microarray images then underwent corrections and normalizations, as specified in a previous protocol ^31^. We selected an intensity cutoff value of Z-score > 9 to produce a significantly reduced list of “positive hits.” Proteins were considered to bind both alleles if the difference between the two allelic averages was less than two standard deviations. If the difference was greater than two standard deviations, then the protein was considered to bind preferentially to either the 11 or 13 DNR allele.

**Electrophoretic mobility shift assays (EMSA).** In order to confirm the differential binding of ELAVL4 and to test the binding of other mTOR signaling components such as S6K, 4EBP and eIF4E we performed EMSA. We utilized the same 3’ fluorescently labeled RNA probes from the microarray, but end-labeled the probes with radioactive γ–ATP-32 to the 5’ end ^32^. The probes were purified (VWR SpinX Filters #29442-754, Clonetech Resin #631770), and precipitated overnight at -80C (3ul yeast tRNA, 6ul, NH_4_Ac, 24ul cold 100% EtOH). Radioactivity of the probe was quantified with a scintillation counter, and each probe was normalized to 4,000 counts per minute (CPM).

The radioactive RNA probes for each of the two DNR alleles were hybridized to commercially purified candidate binding proteins: S6 (Origene #TP317324), 4EBP (NovoProtein #C200), eIF4E (MyBioSource #MBS203380), and ELAVL4 (Origene #TP318612). Each probe was incubated on ice for 20 minutes in the following solution: 2X Probe Buffer (50% glycerol, 1M HEPES, 1M Tris-HCL, 0.5M EDTA, 100mM DTT, DEPC H2O, Bromophenol blue), BSA (10mg/ml, NEB #B9000s), Poly[d(I-C)] (1ug/mL, Roche #10108812001), DEPC H20, γ–ATP RNA probes (1ul at 4000 CPM), and purified proteins (1ug/ul). Each reaction was loaded into a precast 5% TBE gel (Bio-Rad #4565015) and electrophresed in 1x TBE buffer (Bio-Rad #1610733) at 150V for 30 minutes, room temperature. The gel was air dried in clear cellophane (Bio-Rad #1651779) for 3 hours, and then exposed with autoradiography film (VWR # 8294985).

**Western blot analyses**. We obtained cell lysates from the four 13DNR homozygote clones and four targeted 11DNR control clones, using 1X Passive Lysis Buffer (Promega #E1941). Protein was assayed with a colorimetric kit (Bio-Rad #5000001), quantified with spectrophotometry, and lysates were normalized to 20ug total protein.

Protein was heated to 95C for 5 minutes in the following solution: 2x loading buffer (4% SDS, 10% 2-mercaptoethanol, 20% glycerol, 0.004% bromophenol blue, 0.125 M Tris-HCl), H_2_0. The samples were loaded into precast gels (Bio-Rad #4561083) and electrophresed at 120V for 1 hour in 1x running buffer (Bio-Rad #1610732). The gel was then transferred to a PVDF membrane (Bio-Rad #1620177) via the semi-dryelectrophoretic transfer cell (Bio-Rad #1703940) at 15V for 45 minutes.

After transfer, the membrane was blocked in 5% powder milk and 1x TBS buffer (Bio-Rad #1706435) for >1 hour at room temperature. We then probed the membrane using rabbit anti-CRMP2 antibody at 1:5000 (Sigma #C2993) and mouse anti-GAPDH at 1:4,000 (AbCam #ab9485) in 1% milk overnight at 4C. Following wash steps in 1x TBS-T, we probed with mouse (AbCam #ab6728) and rabbit (Thermo #656120) HRP-conjugated secondary antibodies at 1:10,000 in 1% milk for >2 hours at room temperature. After final washes, the membranes were exposed to chemiluminescence for HRP detection (Thermo #32132), and then exposed to autoradiography film.

For Rapamycin exposures, we grew cells in standard media (Gibco dMEM + 10% Fetal Bovine Serum) containing 30nM Rapamycin (SelleckChem #S1039) for 24 hours. After drug exposure, we obtained the cell lysates as described above.

**Immunofluorescence & Imaging** Targeted HEK293 cells carrying the homozygous forms of the 11 and 13 DNR alleles were grown on Poly-D Lysine (Sigma #P6407) coated coverslips in 12-well plates in standard media (Gibco dMEM and 10% Fetal Bovine Serum) at 37C. The media was changed every other day until day four (~75-85% confluency). Cells were washed twice with 1x Passive Buffered Saline, and then fixed in 4% paraformaldehyde for 15 minutes at room temperature. After removal of the paraformaldehyde and washing, the cells were incubated with 1mg/ml wheat germ agglutinin conjugated with Alexa Fluor 488 (Thermo #W11261) at room temperature for 15 minutes. After removal and washing, the coverslips were mounted on slides with mounting media containing DAPI stain (Vectorlabs #H1200). Images were taken on the Nikon confocal microscope (Nikon Eclipse TI-DH).

**RNA sequencing**.

RNA was extracted from the cell pellets using an RNeasy Mini Kit (Qiagen #74104). The RNA was quantified and normalized to 500ng per sample and the samples were then sent to the Hopkins Core Facility. Agilent BioAnalyzer was used for quality control of the RNA prior to library creation, with a minimum RIN of 8.5. RiboMinus™ technology (ThermoFisher Scientific) was used to deplete ribosomal RNA molecules and Illumina’s TruSeq RNA v2 protocol was used to generate libraries. Specifically, total RNA was converted to cDNA and size selected to 150 to 200 bp in length with 3' or 5' overhangs. End repair was performed where 3' to 5' exonuclease activity of enzymes removes 3' overhangs and the polymerase activity fills in the 5' overhangs. An ‘A’ base was then added to the 3' end of the blunt phosphorylated DNA fragments which prepares the DNA fragments for ligation to the sequencing adapters and barcodes, which have a single ‘T’ base overhang at their 3' end. Ligated fragments were subsequently size selected through purification using SPRI beads and undergo PCR amplification techniques to prepare the ‘libraries’. The BioAnalyzer is used for quality control of the libraries to ensure adequate concentration and appropriate fragment size. The resulting library insert size was 120-200bp with a median size of 150bp. DNA sequencing was performed on an Illumina® HiSeq 2500 instrument using standard protocols for paired end 100bp sequencing. As per Illumina’s recommendation, 5% PhiX was added to each lane as a control, and to assist the analysis software with any library diversity issues. Primary Analysis was also performed in our core facility. Reads were processed through Illumina’s Real-Time Analysis (RTA) software generating base calls and corresponding base call quality scores. CIDRSeqSuite 7.1.0 was used to convert compressed bcl files into compressed fastq files.

Other supplementary information

Microarray DNR Probe sequence

| 11DNR (+/- 6bp) | \| tctctcCTTCTCTCTCTCTCTCTCTCTCTCTtttttt \| \| --- \| \| 5’- ucucucCuuCuCuCuCuCuCuCuCuCuCuCuuuuuuu/3Cy5Sp/ -3’ \| \|  \| |
| --- | --- | --- | --- | --- |
| 13DNR (+/- 6bp) | \| tctctcCTTCTCTCTCTCTCTCTCTCTCTCTCTCTtttttt \| \| --- \| \| 5’ – ucucucCuuCuCuCuCuCuCuCuCuCuCuCuCuCuuuuuuu/3Cy3Sp/ -3’ \| |

CRISPR gRNA sequences:

| DNR_gRNA1 | TCTTTTTTTTCCGCCCTAGC |
| --- | --- |
| DNR_gRNA2 | CTTTTTTTTCCGCCCTAGCT |
| DNR_gRNA3 | TTTTTTTTCCGCCCTAGCTG |

CRISPR repair template amplicon primers:

| HekDNR_250_F | TGCCTGAGAGGAAAGGGAGT |
| --- | --- |
| HekDNR_250_R | AATAGCAAGACCAGCGAAGC |

TGCCTGAGAGGAAAGGGAGTGGCTGGCGGCGCATGCGCCACGGTGGCCGACTTGAACCGAGGCTTTTATTGCTGTAGTTTATTTCCACCCCCTTCCCTCCTGTTTCTCTCTCTCCTTCTCTCTCTCTCTCTCTCTCTCTCTTTTTTTTCCGCCCTAGCTGGGGCTGTGTTGGAGGAGAGGAAGAAAGAGAGACAGAGGATTGCATTCATCCGTTACGTTCTTGAAATTTCCTAATAGCAAGACCAGCGAAGC

CRISPR Off-Target PCR Primers:

| OTGprimer1F | TACACCTCCGTTGCCCAGgc |
| --- | --- |
| OTGprimer1R | AGTCTGGCAGGAGTGATGAC |
| OTGprimer2F | CAGTTCCTTAGCAATGCACTGA |
| OTGprimer2R | CATGGGAATTGGCTGAGACC |
| OTGprimer3F | TCCCTCATCTTTGAATGTGTGC |
| OTGprimer3R | CCCATGATGCACCAGAAAGAG |
| OTGprimer4F | AGTCCCCGCCTTCGCTAGgg |
| OTGprimer4R | CAAGGGCTTTTGAGTTTATCGAG |
| OTGprimer5F | CGCTTCTGTAGGGCCTTCAT |
| OTGprimer5R | CCTTCTTGTCGCAGTGGAAC |
| OTGprimer6F | TTCTCCAAGGAAACCAACATGT |
| OTGprimer6R | TATCCATTTGACCAGCCCCT |
| OTGprimer7F | ATTCATCCGCGACATCCTCT |
| OTGprimer7R | GGTGACTGATCCGATGAAGG |
| OTGprimer8F | TCTCAGCTCGACAGTAGTTATTC |
| OTGprimer8R | ACTGCTCTCACTGTCCGAGt |
| OTGprimer9F | CATTCAAGATGCAACACCTACAA |
| OTGprimer9R | CCGTAGTTGAGGGGAGGGgc |
| OTGprimer10F | CACTACATACTGACCGCCAC |
| OTGprimer10R | TGTGATCTGAACTTTTCCAGCC |
| OTGprimer11F | TGTAGGAATCAGAGCCCAATATT |
| OTGprimer11R | AGCAACCTCACTCTTGTCTG |
| OTGprimer12F | GTTGAAGGATGATGGGGTGC |
| OTGprimer12R | GAGCTCTGTCTTCCTCTTCCT |
| OTGprimer13F | CGATTGGTGGCTGACTCATG |
| OTGprimer13R | TGAGAATTGACAGCCCTCCA |
| OTGprimer14F | AAAGGAGAGAAAGGAGATACTGG |
| OTGprimer14R | TTCATGGGCAAAGAATGTTCCC |
| OTGprimer15F | TGTAACATGTTCATCTTCTCCCT |
| OTGprimer15R | GGTGACATAATTCCCATCCGAC |
| OTGprimer16F | GATTTCCAGATGCATGGGTCC |
| OTGprimer16R | TGGCTTTTCTTCCAACAGGA |
| OTGprimer17F | GTGGGTGACTGCTTGACTTT |
| OTGprimer17R | GACATGCCAAAAGGCCCTTG |

RT-PCR Primers

DPY_clip_1F: TCCCGGCAGTTTTTGCCTTA

DPY_clip_1R: AGACGATCGCTCGTGATGC
